# Author profiles in Web of Science, Scopus, Dimensions, ORCID, ResearchGate, and Google Scholar: a case report

**DOI:** 10.5195/jmla.2026.2304

**Published:** 2026-10-01

**Authors:** Kimberly R. Powell

**Affiliations:** 1 krp0069@auburn.edu, Health Sciences Librarian, Auburn University Libraries, Auburn University, Auburn, AL

**Keywords:** Web of Science, Scopus, Dimensions, ORCID, ResearchGate, Google Scholar, author identifiers, author disambiguation, researcher impact

## Abstract

**Background:**

This study aimed to benchmark the availability and accuracy of author profiles for 45 faculty members within the Auburn University College of Nursing. Profile availability in the database platforms Web of Science, Scopus, and Dimensions were analyzed alongside the author-driven platforms ORCID, ResearchGate, and Google Scholar to set an understanding for the potential efforts needed for profile curation, publication tracking, and research impact assessments moving forward.

**Case Presentation:**

Each of the six platforms were manually searched for faculty and name variations alongside both current and past institutional affiliations. All identified profiles were collected. Associated publication counts were recorded and database results were screened for accurate author identification. Among the author-driven platforms, ORCID had the highest adoption rate, with 64% of faculty, while ResearchGate and Google Scholar trailed with 38% and 16% faculty adoption, respectively. Web of Science offered profiles for 82% of faculty; Scopus offered profiles for 80% of faculty, and Dimensions only 53%. However, 53% of the associated publications were misattributed in Web of Science profiles compared to just 1% misattribution in Scopus and none in Dimensions.

**Conclusions:**

Among our faculty, Scopus offered the most efficient author profile platform, balancing profile availability, minimizing misattribution, and including avenues for early identification of new authors. While largely in line with previous study findings, this effort provided important contextual understandings of profile data availability which can be used to inform research impact assessments and library services supporting author profiles for highlighting scholarly influence.

## BACKGROUND

In recent years academic librarians across disciplines have become more involved in author tracking, curation of author profiles, and publication reporting (1–4). As experts in bibliographic databases, librarians are well positioned to offer specific support in using and managing database-driven profiles available in literature databases such Web of Science and Scopus. These bibliographic databases automate author profile creation as part of the bibliometric discovery features. Author-driven profiles available through ResearchGate and Google Scholar have also come to be widely acknowledged and recommended for increasing research visibility (5–8). These author-driven profiles are website platforms in which individuals elect to create and maintain research and publication profiles outside of the curated literature.

Database-driven author profile automation was first introduced by Scopus in 2006 (9). Profile creations are automated based on listed author name, email, institutional affiliation, and patterns of citations, co-authorship, and subject domains (10). Web of Science followed suit with their Researcher ID, which launched initially as an author-driven identifier in 2008 (11). In 2019, Web of Science moved its Researcherr ID profiles to primarily machine-learning driven methods (12). While profiles are automatically created and publications are automatically added, individual researchers are still able to claim or verify their profiles, as well as merge duplicate accounts and submit corrections to identified records and name variations. The Dimensions database was launched in 2018 with researcher profiles included in the freely available publications module. Uniquely, Dimensions profiles provide automated linking to public Open Researcher and Contributor ID (ORCID) profiles (13). Notably, Web of Science and Scopus also make their author profiles, including searching, claiming, and managing (including connections with ORCID), freely available without need for full database subscriptions or licensing (10, 14).

In addition to database-driven author profile algorithms, web-based platforms like ResearchGate and Google Scholar offer authors a chance to gather and highlight their work on public web pages by creating individual user accounts. Once authors create an account, both platforms will add or suggest additional records, including non-traditional record types and citations scraped from web content such as abstracts, datasets, thesis, syllabi, etc. ResearchGate launched in 2008 as a social connection platform and remains popular for full-text document sharing (6). Google Scholar user profiles, launched in 2012, is another popular choice, particularly for those looking to capture high citation counts and activity as a result of the non-traditional citation tracking such as those made by abstracts, datasets, thesis, dissertations, syllabi, and personal webpages (15, 16). Neither of these two profile sites offer third party curation options or interoperability with other platforms; these are stand-alone platforms and cannot currently be linked with outside platforms.

Since launching in 2012, ORCID sits in a space between the automated database author identifiers and the author-driven webpages. As an author-driven profile system, researchers must choose to create and maintain an account. However, individuals can also allow delegates or trusted organizations, including literature databases and universities, to write data to their ORCID record. The ORCID platform has gained recognition as the unique author identifier required for an increasing number of federal funding compliance and data security guidelines (17, 18). All three of the major bibliometric databases, Web of Science, Scopus, and Dimensions, allow for ORCID integrations. In the case of Dimensions, ORCID iDs are automatically linked to the researcher profiles created by the platform. In Web of Science and Scopus, researchers have the option to link their ORCID iD to their researcher profile in the database once they have claimed their profile. Claiming a Web of Science or Scopus created profile also allows the researcher to verify or reject publications linked to their profile.

Faculty authors have a variety of platforms to choose from when trying to collect and showcase their research and scholarly outputs. Universities and research organizations face similar challenges when trying to track and aggregate their researchers’ efforts. Librarians are natural partners when looking to collect and assess the available literature, but the accuracy of available author profiles across platforms has not yet been fully compared or assessed.

### Case Presentation

The case presented here aimed to evaluate the availability and accuracy of author profiles for a convenience sample of the 45 faculty members which made up the 2024 Auburn University College of Nursing (AUCON) faculty. In 2019, the university was recognized with the highest research classification (R1) Carnegie Classification status, denoting it as a doctoral institution with very high research output (19).

Relatedly, AUCON has shown significant research growth in recent years, and in 2024 was one of three nationally ranked programs at Auburn by US News and World Report (20). The 2024 university strategic plan articulates a continued commitment to research growth with a goal to double publication outputs by 2034 (21).

Even prior to these formal research recognitions and strategic goals, the library has been increasingly called upon to provide expert publication tracking and affiliation searching in support of institutional publications assessments. Since 2022, the library has offered ORCID profile support and tracking, helping to ensure affiliated researchers are meeting federal and publication requirements [see more: https://libguides.auburn.edu/ORCID]. However, prior to 2025, there had not been any campus support of author-level profile systems outside of ORCID. When consulted, librarians within the institution would recommend Web of Science or Scopus for publication assessments, as these both are included in institutional subscriptions. However, Dimensions has also been used in recent assessment projects and licensed through the Provost’s office. Similarly, an expert finder project launched in 2025 uses profiles available through Google Scholar (see more: https://ausme.auburn.edu/). As the library is becoming more involved in the analysis of and recommendations for author profiles, this study sought to benchmark faculty profile availability across six platforms to inform potential profile curation and publication tracking efforts moving forward.

### Profile Identification

A total of six platforms were searched for affiliated author records to 45 nursing faculty members, including name variations and previous affiliations. These platforms were: Web of Science (free profile URL: https://www.webofscience.com/wos/), Scopus (free profile URL: https://www.scopus.com/freelookup/form/author.uri), Dimensions (free database URL: https://app.dimensions.ai/auth/base/landing), Google Scholar (https://scholar.google.com/), ResearchGate (https://www.researchgate.net/), and ORCID (https://orcid.org/). When available, the unique profile ID was collected. Additionally, it was noted if the profile was “claimed” or “verified” by the represented author, and if the profile had been linked to an ORCID profile. For Google Scholar and ResearchGate, which do not assign a unique identifier, the direct URL was collected in its place.

A spreadsheet was created for all nursing faculty members listed in the institutional directory, which included tenured, tenure-track, and clinical faculty. Author profiles were then manually collected from each of the platforms in October 2024. Columns were added to the spreadsheet to track identified profile ids and URLs. Profile identification was completed in an iterative process as name variations, affiliations, and record details were discovered in subsequent platform searches. A second review was completed in April 2025 to capture additional profiles, verification status changes, or ORCID links through the 2024 calendar year. Several additional author profile accounts were uncovered at the time: 1 ORCID profile, and 3 additional author profiles in Dimensions.

As of April 2025, a total of 192 author profiles were collected for the 45 AUCON faculty. Table 1 shows the identification of faculty associated profiles across each platform. Overall, 40 faculty members (89%) had a profile on at least one platform, with 38 (84%) appearing with at least one database-driven profile. There were 19 (42%) unique authors represented with an author-driven profile on either Google Scholar or ResearchGate platforms. Only 6 (13%) authors appeared with profiles across all platforms. Among the 5 individuals with no profile on any platform, all were assistant ranked clinical faculty who are less likely to have formal publication records than tenured, tenure-track, or later career (associate and full ranked) faculty. However, these five make up only 19% of the 26 total assistant clinical faculty included in the sample.

**Table 1:** Summary of author profile availabilities.

	Web of Science	Scopus	Dimensions	ORCID	Google Scholar	ResearchGate
faculty with no available profile	8	9	18	15	38	28
faculty with 1 associated profile	22	28	19	30	7	16
faculty with 2 associated profiles	11	5	8	0	0	0
faculty with 3 associated profiles	4	3	0	0	0	1
**unique faculty with available profiles**	**n=37**	**n=36**	**n=27**	**n=30**	**n=7**	**n=17**

Of the database-driven platforms, faculty most frequently appeared with Web of Science Researcher IDs (n=37, 82%), followed closely by Scopus author profiles (n=36, 80%).

Dimensions offered profiles for the least number of faculty (n=27, 60%). All database platforms had multiple associated profiles for at least one faculty author. The problem of multiple associated profiles was most pronounced in Web of Science where 15 (41%) of the available faculty in the database were represented under more than one identifier.

For author-driven profiles, faculty most frequently appeared in ORCID (n=30, 67%) followed by ResearchGate (n=17, 38%) and lastly Google Scholar user profiles (n=7, 16%). No profile duplication was found on ORCID or Google Scholar. One individual had 3 ResearchGate accounts under their name and institutional affiliation, but none were associated with any publication records or data.

#### Profile claiming

Faculty were most likely to have claimed their respective Scopus profiles (n=8, 22%) as represented by profiles manually linked with an ORCID iD. Available Web of Science profiles showed only 3 (8%) with a linked ORCID iD among the total of 6 (16%) claimed records. While Dimensions offered the lowest availability of author profiles, it also offered the highest proportion of profiles linked to ORCID profiles (n=12, 50%). ORCID linking is a uniquely automated process for Dimensions, with the ORCID linking on this platform done without requiring author verification or claiming. Individuals are not able to claim, verify, or modify author accounts themselves within the Dimensions database as they can on the other platforms (22). Figure 1 illustrates the proportion of researchers with verified or ORCID linked profiles within each of the database platforms.

**Figure 1:**
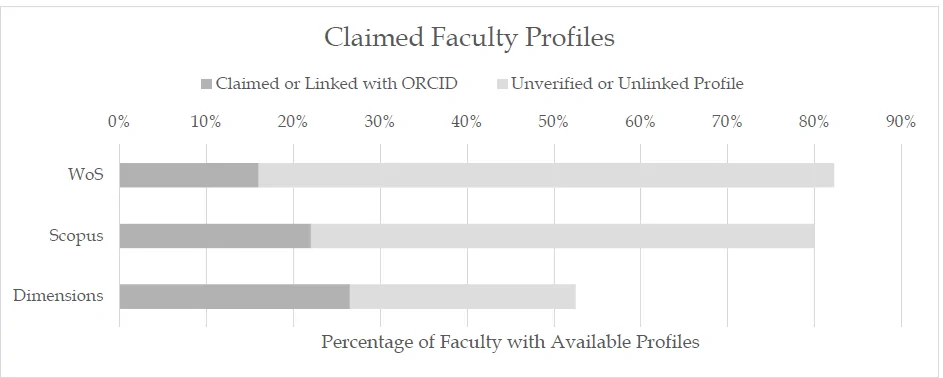
Percentage of author profiles in each database with breakout of those claimed or linked with ORCID.

#### Publication record completeness

Publications affiliated with each of the Web of Science, Scopus, and Dimensions profiles were collected in April 2025 and saved to Endnote groups (version 21.5, Clarivate Analytics) for deduplication and manual verification of correct authorship attributions. When authorship could not be confirmed by the available record metadata, internally provided CVs were consulted. Exports from Scopus and Web of Science were limited to articles, books, book chapters, conference/proceedings papers, and reviews. Publication records were deduplicated using both manual and automated features to identify unique indexing across the database and create a total unique count of publication records for each faculty author. Dimensions was the only database to return preprints & conference and poster abstracts, which were removed manually. The remaining unique publications attributed to each author, gathered across these three databases, were used to calculate the percentage of database coverage.

For ORCID, ResearchGate, and Google Scholar only the count of claimed publications was recorded, with no effort made to verify authorships. Each of these platforms allow for expanded record types such as abstracts, posters, datasets, and preprints which do not easily allow for independent verification and were explicitly excluded from the collected database publications. For identified ORCID profiles, it was further noted if data were set to private or public view. If public, the number of listed works/publications and current affiliation availability were also noted.

A unique total of 363 verified publication records were collected across all database author profiles (Web of Science, Scopus, and Dimensions), with publication years ranging from 1979 through 2024. As author profiles are typically intended to offer tracking across the full spectrum of a researcher’s career, these counts include all authored records regardless of institutional affiliation at the time of publication.

The Dimensions platform returned 318 publications records associated with the identified author profiles; 28 of these records were uniquely indexed in Dimensions and not available through Web of Science or Scopus. An additional 31 records were later identified as available in Dimensions, but the listed institutional author was not associated with an available author profile. Within Dimensions, no publications were misattributed to their respective author profiles. Scopus also returned 318 publication records associated with their author profiles, with 4 uniquely indexed in Scopus. There were 2 misattributed publication records, both associated with the same profile, which were removed. Web of Science returned 513 publication records. However, author misidentification was highest here, with 53% (n=271) of associated records being misattributed to their listed author profile(s). The majority of misattributions appeared to be due to the conflation of authors with similar surnames and initials working in related fields. After removing these erroneous records, Web of Science captured 242 faculty publication records. None of the identified records were unique to Web of Science. The percentage of the total verified faculty publications within each of these database profiles is displayed in Figure 2a (left), while Figure 2b (right) showcases the extent of publications erroneously attributed to the respective author profiles in each database.

**Figure 2:**
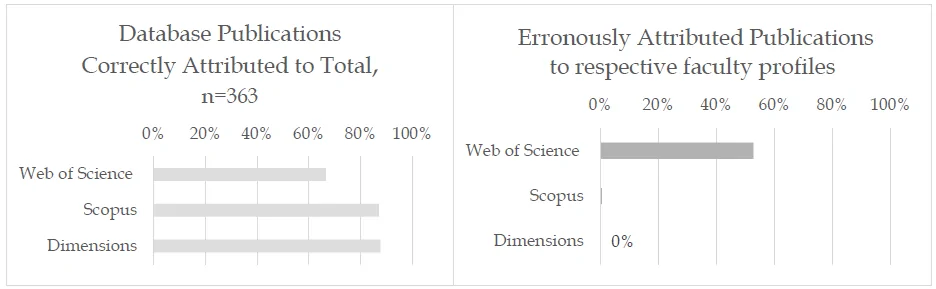
Authorship attribution within each database. A) The percentage of total identified publications correctly attributed by database profiles. B) The percentage of publications in each database erroneously attributed to collected author profiles.

While 67% (n=30) of faculty had an identifiable ORCID iD, 4 of these had no data publicly available in the account. Additionally, 8 ORCID profiles did not include any authored publications, despite having a combined authorship count of 95 publications for these authors identified from the database profiles. Across all 45 faculty members, 27% (n=12) had an ORCID profile with at least 75% of their total publication count listed. ResearchGate similarly provided a mean of 72% of the total publication count for participating authors. However, this is likely an inflated estimate as ResearchGate records include preprints, data files, meeting abstracts, and other record types not included in the counts identified through Dimensions, Scopus, or Web of Sciences. While the expanded record types can be controlled for in ORCID’s associated “type” data for each record, they cannot be controlled for through ResearchGate or Google Scholar associated metadata.

Google Scholar offered a mean 63% increase over the database identified records for each author. The highest profile discrepancy had 22 identified publications from the database-driven profiles and 51 listed records in Google Scholar, a 132% increase. While reconciliation of the Google Scholar listing is outside the scope of the present efforts and reporting aims, many of these additional records appeared to be abstract, poster, and conference listings excluded from the collected database counts.

## DISCUSSION

Tracking author profile adoption and associated publication records is an important exercise in understanding the discoverability and potential measures of scholarly impact. Benchmarked data, such as presented here, give insight into faculty output representations over time as well as an awareness of potential data cleaning and curation efforts needed before author profile platforms can be reliably used for highlighting faculty efforts and institutional reporting. The current documentation of author profiles at our institution informs potential patterns in the availability of author assessment data across the various platforms and underscore the continued limitations of author-driven profiles such as ORCID for assessment purposes.

Among the author-driven profiles, AUCON faculty had the highest adoption of ORCID profiles. This is likely linked, in part, to campus outreach and curation efforts following US federal funding requirements and the National Security Presidential Memo 33 (NSPM-33) recommendations for persistent identifiers (23, 24). ORCID adoption by Auburn college of nursing faculty is slightly lower than a recent broader report of 31 academic institutions which showed an overall faculty adoption of 72% across research disciplines, and 85% adopt in “health professions and related clinical services” (17). However, even for those authors that do have public ORCID profiles, robustness in both studies still falls short of being able to use faculty ORCID profiles for reliable publication metrics or institutional reporting. The average AUCON profile reported less than three-quarters of the available publication records and less half of faculty could be discovered by current affiliation listings. These missing elements are crucial impediments to accurate and robust impact reporting and assessments severally undermining the potential of using ORCID for local assessments.

Fortunately, Web of Science, Scopus, and Dimensions platforms all allow for data sharing with linked ORCID accounts. Library services and institutional efforts around supporting more complete ORCID data or publication reporting can leverage these connections to increase discoverability and inclusion of identified publications in author impact measures and reporting analytics. Neither ResearchGate nor Google Scholar allow for ORCID connection or integration.

Following ORCID, Auburn nursing faculty were most likely to have created ResearchGate profiles, which follows trends previously reported in the literature (2, 3, 25). Early evaluations of ResearchGate highlighted the potential influence of academic social networks to disrupt existing hierarchies, allowing earlier career researcher and those from the Global South to better highlight their scholarly works, but more recent studies have largely found the dominate hierarchies to be reinforced (26). Studies among university faculty have since found document sharing to be a leading motivation for ResearchGate participation (27, 28). Notably, ResearchGate provided metrics were not generally seen as an accurate reflection of research impact and were, in fact, discontinued in 2022 (29, 30).

Among the database-driven platforms, Scopus seemed to offer the best balance of accuracy and efficiency for Auburn nursing faculty. This is in line with previous findings comparing and assessing the representation of nursing publications and impact measures (31–33). Among the three automated profile platforms examined here, Scopus outperformed Dimensions in the availability of automated faculty profiles. Scopus also outperformed Web of Science for faculty-claimed or ORCID linked profiles and tied with Dimensions for the highest count of identified publications. Scopus also offered third-party correction submissions, with a specific toggle for librarian curation efforts, a feature likewise available in Web of Science, but not available in Dimensions. All Web of Science and Scopus corrections uncovered as a result of the analysis presented here were processed and available in their respective platforms within seven days of librarian curation submissions. Like the profiles themselves, claiming, linking, corrections and modifications are accessible in Web of Science and Scopus regardless of institutional subscription access.

This investigation also uncovered that database-driven profiles may have important implications for early career researchers that was not found to be reported elsewhere. Notably, Dimensions does not provide author profiles or assign author identifiers until two associated records are available. The finding of associated publications available in the database yet unlinked with discoverable author profiles does echo previous findings with affiliation profiles. Guerrero-Bote et al. (2020) noted in their study that over 25% of Dimensions records remained unlinked to institutional affiliation profiles (34). Conversely, Scopus does assign an identifier to single-record authors, but these are only discoverable from the publication record itself; single-record author profiles will not be returned in author search results lists. In yet another approach, Web of Science profiles allow individuals to create accounts prior to publications records but were also found the most likely to append erroneous records to these profiles resulting in misattributed publications.

## CONCLUSIONS

The comparisons discussed here are a case study offering a snapshot of author profile adoption and database accuracy in a single faculty setting at one institution. While many of the findings are in line with previous reports from the literature, the profile identification and verification exercise was an essential effort in understanding the local faculty representations. This lays the groundwork for further institutional assessment recommendations. The comparisons discussed here offer greater contextual understandings when recommending platforms, and the associated curation efforts and time allocations for supporting librarians.

Among our faculty, Scopus offered the most efficient automation of associated author profiles, balancing profile availability, minimizing misattribution, and including avenues for early identification of new and early-career authors. As evidenced by growing federal and publisher support, ORCID iDs have tremendous potential to incorporate author-driven representation as well as integration across the database platforms. However, its practical application for author assessments remains ineffective due to low adoption and incomplete publication data. At Auburn University, campus initiatives have been launched relying on Google Scholar available profiles, and ResearchGate still remains relatively popular among our faculty authors. While these have been also shown to be inadequate platforms for assessing faculty authorship profiles, they cannot be discounted. Similarly, Dimensions offered a high level of associated record accuracy as well as the automated ORCID linking, making it an important emerging resource that many librarians and information professions should consider for exploring their own author contexts.

## Data Availability

The data associated with this article are available in the Open Science Framework (OSF) at https://osf.io/ypa76.
